# “If they take it without knowing, they will default…”: perceptions of targeted information transfer to promote adherence to intermittent preventive treatment with dihydroartemisinin-piperaquine for the prevention of malaria in pregnancy in western Kenya

**DOI:** 10.1186/s12936-024-05131-6

**Published:** 2024-11-29

**Authors:** Jenna Hoyt, Hellen C. Barsosio, Isdorah A. Odero, Benson Omondi, Florence Achieng, Simon Kariuki, Jenny Hill, Jayne Webster

**Affiliations:** 1 Department of Clinical Sciences, Liverpool School of Tropical Medicine, Liverpool, UK; 2 Kenya Medical Research Institute, Centre for Global Health Research, Kisumu, Kenya; 3 Disease Control Department, London School of Hygiene and Tropical Medicine, London, UK

**Keywords:** Intermittent preventive treatment, Malaria in pregnancy, Dihydroartemisinin-piperaquine, Adherence

## Abstract

**Background:**

Increasing resistance to sulfadoxine-pyrimethamine (SP) threatens the effectiveness of intermittent preventive treatment (IPTp) to prevent malaria in pregnancy. Dihydroartemisinin-piperaquine (DP) is the most promising candidate to emerge from clinical trials, but requires a multi-day regimen. Despite being a single-dose regimen, coverage of IPTp-SP remains low, fuelling concerns about adherence to multi-day drug options. An implementation feasibility trial in routine antenatal care settings in western Kenya demonstrated that adherence to the multi-day DP regimen was improved when IPTp-DP was delivered with a targeted information transfer intervention that comprised healthcare provider training and communication tools to support delivery and uptake. This study explored healthcare provider and pregnant women perspectives to understand (1) how the targeted information transfer improved adherence to IPTp-DP and (2) if improved adherence to IPTp-DP influenced provider perceptions towards implementation feasibility of multi-day drug regimens for IPTp.

**Methods:**

In-depth interviews were conducted with 64 healthcare providers and 64 pregnant women, selected using a convenience sampling approach from across the three trial arms: IPTp-DP+ (with intervention), IPTp-DP, and current standard of care IPTp-SP. Transcripts from healthcare providers and pregnant women were coded in Nvivo-12 using separate a priori frameworks that included components of the consolidated framework for implementation research. Thematic analysis was used to understand how the targeted information transfer affected adherence to IPTp-DP and how concerns about adherence might influence provider perceptions towards multi-day drug regimens for IPTp.

**Results:**

Adherence to IPTp-DP doses taken at home was compromised when women experienced unpleasant side effects. Pregnant women valued being given information about IPTp-DP, including potential side effects and how to manage them. Among providers in the IPTp-DP + arm, confidence in advising women on how to manage side effects increased, and they believed this guidance improved adherence. When concerns about adherence were reduced, providers in the IPTp-DP + arm were positive about implementation feasibility, whereas providers in the IPTp-SP arm remained focused on the dosing complexities and were less convinced of the feasibility of implementing IPTp-DP.

**Conclusions:**

Healthcare provider confidence in advising women on how to minimize side effects was boosted through targeted information transfer, which was perceived to improve adherence to IPTp-DP. Policy makers are encouraged to consider supportive interventions that enhance provider confidence around adherence should they shift to multi-day drug regimens for IPTp.

**Supplementary Information:**

The online version contains supplementary material available at 10.1186/s12936-024-05131-6.

## Background

Malaria in pregnancy is associated with adverse health outcomes for both mother and baby, including severe maternal anaemia, miscarriage, stillbirth, premature birth and low birthweight [1]. In sub-Saharan Africa, low birth weight resulting from malaria infections during pregnancy is responsible for approximately 100,000 infant deaths per year [2]. A comprehensive package of preventive measures recommended by the World Health Organization (WHO) includes the use of long-lasting insecticidal nets and intermittent preventive treatment (IPTp) with sulfadoxine-pyrimethamine (SP), routinely delivered through the antenatal care (ANC) platform, alongside passive case management [3]. In Kenya, IPTp-SP is administered in malaria endemic areas as a single dose via directly observed therapy (DOT) at every ANC visit from the second trimester, with doses given at intervals of at least four weeks [4]. However, with evidence of the reduced effectiveness of SP due to widespread resistance [5], alternative therapies are urgently needed. As a promising candidate to replace SP for IPTp, dihydroartemisinin-piperaquine (DP) has a good safety profile [6], is well tolerated by pregnant women, and has a long prophylactic effect [7, 8]. Results from recent clinical trials indicate monthly regimens of DP are most effective at reducing malaria parasitaemia [6, 7, 9]. However, replacing the inexpensive, single-dose SP regimen with a more complex, multi-day artemisinin-based combination therapy (ACT) could present implementation and uptake/adherence challenges. As such, it is critical to assess the implementation feasibility prior to any policy decision on IPTp-DP [10].

IPTp-DP was found to be acceptable to both healthcare providers and pregnant women in the context of a clinical trial in western Kenya, but concerns were raised about adherence under routine conditions [11]. There is no available evidence on the implementation feasibility of IPTp-DP. Feasibility studies in Kenya have explored the use of DP with intermittent screening and treatment (ISTp) – where pregnant women are treated with DP if they test positive for malaria. One study found that whilst DP was well tolerated by pregnant women, healthcare providers expressed concerns about adherence to the multi-day drug regimen [12], which confirms findings from earlier acceptability studies [11, 13]. This concern was demonstrated in another study where only 6% of women received adequate counselling on how to take the remaining DP doses at home, leading to recommendations of enhanced healthcare provider training to improve counselling practices [14]. Adherence to ACTs for treatment of malaria varies across settings but the evidence is limited [15]. Visual aids alongside provider instructions have been found to improve adherence to ACTs among children and adults in Malawi [16] and India [17].

Barriers to optimal coverage of the current IPTp-SP policy are well documented [18]. Dislike of taking drugs during pregnancy and unpleasant side effects are well-established barriers to IPTp-SP uptake [18, 19] and could similarly hamper adherence to DP. Conversely, if DP is better tolerated or perceived as a more effective antimalarial than SP, demand for IPTp-DP may increase. This study was nested in a three-arm pragmatic cluster randomized controlled trial (cRCT) exploring the feasibility of IPTp-DP with or without targeted information transfer that comprised additional provider training and the provision of communication tools to support delivery, uptake and adherence, versus standard of care (IPTp-SP) in routine ANC settings in western Kenya [20]. In the cRCT, adherence was defined as the proportion of pregnant women visited at home who reported completing their 3-day DP regimen for their most recent dose as prescribed, verified by pill count. Relative to IPTp-DP only, adherence in the IPTp-DP arm with targeted information transfer was 16% higher (aRR) = 1·16, 95% CI 1·03 − 1·31; *p* = 0·0140 [20]. In this study, we explored how the targeted information transfer improved adherence to IPTp-DP doses taken at home and, consequently, if the improved adherence to IPTp-DP influenced healthcare provider perceptions towards the implementation feasibility of multi-day IPTp drug regimens.

## Methods

### Study design

This qualitative study was nested in a pragmatic 3-arm cRCT (NCT04160026) to understand how the targeted information transfer influenced adherence to DP and the feasibility of delivering IPTp using multi-day drug regimens. Pregnant women received either IPTp-DP with targeted information transfer (IPTp-DP+) or without (IPTp-DP) versus standard of care (IPTp-SP). Full details of the trial and the intervention design are described elsewhere [20]. This study explored how targeted information transfer affected pregnant women’s adherence to IPTp-DP  based on the perspectives of healthcare providers delivering, and of pregnant women receiving, the intervention, compared to those in the other arms. In addition, the study explored the role of adherence on healthcare provider beliefs and attitudes towards delivering IPTp-DP, a multi-day regimen, versus the current policy of single-dose IPTp-SP delivered in the health facility by DOT. The findings of this study are reported in accordance with the Standards for Reporting Qualitative Research (SQRS) guidelines for qualitative research (Supplementary file 1).

### Study sites and context

This study was conducted in western Kenya in the malaria-endemic counties of Kisumu and Homa Bay at health facilities adjacent to the clinical trial sites. According to the 2020 Kenya Malaria Indicator Survey [21] 67.7% of pregnant women in the endemic lake zone, where Kisumu is situated, attended between four and seven ANC visits in their last pregnancy, and over 97% receive ANC from a skilled provider. Uptake of at least one dose of IPTp-SP was 79.9% and uptake of three or more doses increased from 35% in 2015 to 48% in 2020.

### Intervention

The three preventive treatment interventions were delivered in 18 randomly selected ANC clinics, six per arm, delivering routine ANC services over a period of 10 months. However, three facilities were dropped mid-trial after being repurposed to COVID-19 treatment centres and stopped providing ANC services, including IPTp. Since one facility per arm was dropped, this did not bias the study. Healthcare providers were trained to deliver IPTp-DP in the 10 intervention facilities or given refresher training on IPTp-SP in the five control facilities. As such, HIV-uninfected pregnant women attending the study facilities in the 2nd or 3rd trimester received either (1) monthly IPTp-SP: standard of care administered as a single dose of SP by DOT, (2) monthly IPTp-DP: a 3-day course of DP (3–5 tablets per day based on bodyweight) or (3) monthly IPTp-DP+: IPTp-DP with the targeted information transfer intervention. The targeted information transfer intervention, introduced five months after IPTp-DP was in use, included a package of communication tools such as job aids to assist providers with the weight-based DP regimen and visual aids to support the counselling of women around issues related to adherence. For instance, women were given instructions on the dosing schedule and information about potential side effects they might experience and how to manage them (e.g., take DP doses at night before going to bed). Women needed to return to the health facility for subsequent ANC visits in both the 2nd and 3rd trimester to receive further doses of IPTp (SP or DP) at monthly intervals. Further details on the intervention, including the visual aids used are published in the trial evaluation [20].

### Study participants and procedures

Study participants included pregnant women attending ANC and receiving IPTp drugs and healthcare providers delivering IPTp drugs, in each of the three trial arms. In-depth interviews (IDIs) were conducted between June 24 and September 11, 2020, approximately eight months post-implementation of IPTp with DP and three to five months after the targeted information transfer intervention was introduced. At each of the 15 health facilities (five per intervention arm) between 2 and 5 pregnant women and healthcare providers were selected. Participants were selected using a convenience sampling approach, that is women and providers who were present and available on the day at the facility, were invited to participate. This approach was employed primarily due to budgetary and time constraints in the study.

IDI topic guides were developed to elucidate healthcare provider perceptions on (1) IPTp-DP and IPTp-DP + versus the standard of care (IPTp-SP), (2) adaptations to their working practices that would be needed to implement IPTp-DP or IPTp-DP + should it become policy, (3) their recommendations to ensure effective implementation in their health facility, and (4) their perceptions on the feasibility of implementing IPTp-DP or IPTp-DP + at scale. Topic guides used for IDIs with pregnant women explored (1) experiences with IPTp (either DP or SP depending on arm), (2) acceptability of IPTp and of either DP or SP, (3) information about IPTp and DP or SP received at ANC, and (4) adherence to DP for women in the IPTp-DP/DP + arms. Interviews were carried out by two trained interviewers (one male and one female) and conducted in English with healthcare providers and either Kiswahili, Dholuo or English with women based on the participant’s preference. The interviews were audio recorded and then transcribed and, where necessary, translated to English. Audio files and transcripts were labelled using the participant ID number, stored in a secure location and accessed only by authorized members of the research team to ensure confidentiality.

### Data management and analysis

Transcripts from the IDIs were imported into NVivo-12 (QSR international) for coding and analysis. Transcripts were labelled by arm (DP, DP + or SP), county, participant ID, participant category (healthcare providers or pregnant women), and date of interview. Coding of the transcripts was carried out by one researcher (JHo) and coding validation sessions with the field team were conducted to ensure correct interpretation of the data. Transcripts from IDIs with providers and pregnant women were inductively coded separately around a priori frameworks that both comprised (1) general context (burden of malaria, ANC visits, health facility structure), (2) selected constructs from the Consolidated Framework for Implementation Research (CFIR) [22], and (3) key components of IPTp (separated by arm DP, DP + and SP) that included dosing, schedule, mode of delivery, information on IPTp given by healthcare providers, side effects, and adherence to DP, including factors that facilitate and block adherence.

The CFIR is widely used in implementation research to guide the assessment of implementation contexts and identify factors that influence intervention implementation. It consists of 38 operationally defined constructs within five domains [22]. Themes were coded to the CFIR using a ‘menu of constructs’ approach – which allowed for the selection of relevant constructs rather than using the framework as a whole [23]. The CFIR constructs were selected, and the construct definition was adapted to reflect the three arms (IPTp-DP, IPTp-DP + and IPTp-SP) implemented in this study and the different participant groups (pregnant women and healthcare providers). This exercise was undertaken prior to coding and was based on a discussion among the researchers in relation to what was known about the interventions from the literature and considering the participant groups. Constructs for healthcare providers were selected from all five CFIR domains (intervention characteristics, outer setting, inner setting, characteristics of individuals and, process) whilst only three of the five domains (intervention characteristics, outer setting and, characteristics of individuals) were deemed relevant to pregnant women and therefore included in the coding framework (Table 1).

**Table 1:** CFIR domains, constructs and adapted definition selected for analysis by participant group

Domain	CFIR construct	PW/HP	Definition of construct used to guide coding
Intervention characteristics	Relative advantage	PW/HP	Perceptions of the advantages or disadvantages of using IPTp with DP vs. the current policy of IPTp with SP → includes perceptions of DP vs. SP
	Adaptability	PW/HP	Does IPTp with DP need to be adjusted to make it more palatable to PW → includes changes to the drug, dosing, and schedule
	Complexity	PW/HP	Perceptions on the difficulty of implementing ITPp with DP → includes the challenges with regards to DOT and adherence
	Costs	HP	Costs associated with implementing IPTp with DP → includes the financial costs and sustainability of DP
Outer setting	Patient’s needs & resources	PW/HP	How PW feel about different elements of the intervention → includes HP perceptions on PW acceptability towards IPTp and DP → includes HP perceptions on how information is best delivered to PW
	External policy & incentives	HP	Perceptions on what is required at the policy level to implement the intervention
Inner setting	Tension for change	HP	Perceptions on the challenges of the current IPTp with SP strategy → includes SP resistance
	Compatibility	HP	How the intervention is perceived to fit into the existing system → includes ease/challenges with delivery of IPTp with DP
	Leadership engagement	HP	Who would need to be involved in implementing the new intervention → at sub-county and national levels
	Available resources	HP	What resources are required to deliver IPTp with DP → includes costs, drug availability, health information, time, and training
	Access to knowledge & information	HP	What information is available to support HP in delivering IPTp with DP/SP → includes perceptions of job aides
Characteristics of individuals	Knowledge & beliefs about intervention	PW/HP	Attitudes toward and value placed on IPTp with DP → includes perceptions of the benefits of using DP
	Self-efficacy	PW/HP	Beliefs about their capacity to deliver/take IPTp with DP → includes perceptions of IPTp with DP as a burden
Process	Engaging	HP	Who needs information about IPTp with DP prior to implementation → includes sensitisation at the community level & with PW → includes training requirements for HPs and CHVs
	Executing	HP	What needs to be considered to have a smooth transition to IPTp with DP → includes possible challenges to consider

PW – pregnant women, HP – healthcare provider

**Fig. 1:**
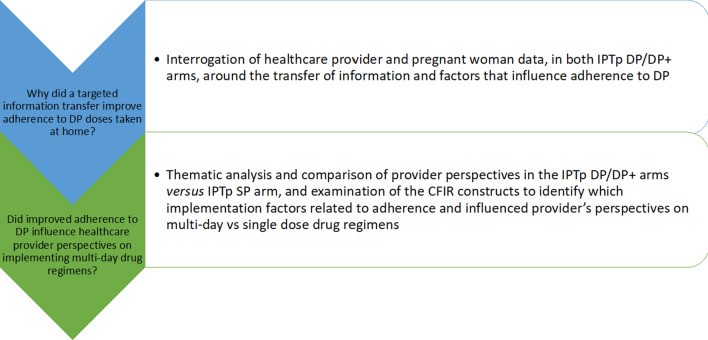
Data analysis by research question

Coded data from both participant groups were extracted and interrogated to address the two specific research questions **(**Fig. 1). This involved examining and comparing themes and sub-themes between the IPTp-DP and IPTp-DP + intervention arms (from both providers and pregnant women) to identify factors that improved (or deterred) adherence to DP doses taken at home and how these themes may have differed between arms. In addition, themes coded to the CFIR constructs were extracted to examine differences in healthcare provider perspectives across all three intervention arms towards single *versus* multi-day drug regimens and, specifically, how concerns about, and experiences with, adherence to DP influenced their viewpoint. Themes and sub-themes were discussed among the research team to ensure meanings reflected the local contexts and to seek consensus where viewpoints differed. Quotes are presented to illustrate key themes and are labelled to reflect the participant group/role, study arm and key charateristics whilst ensuring anonymity.

## Results

**Fig. 2:**
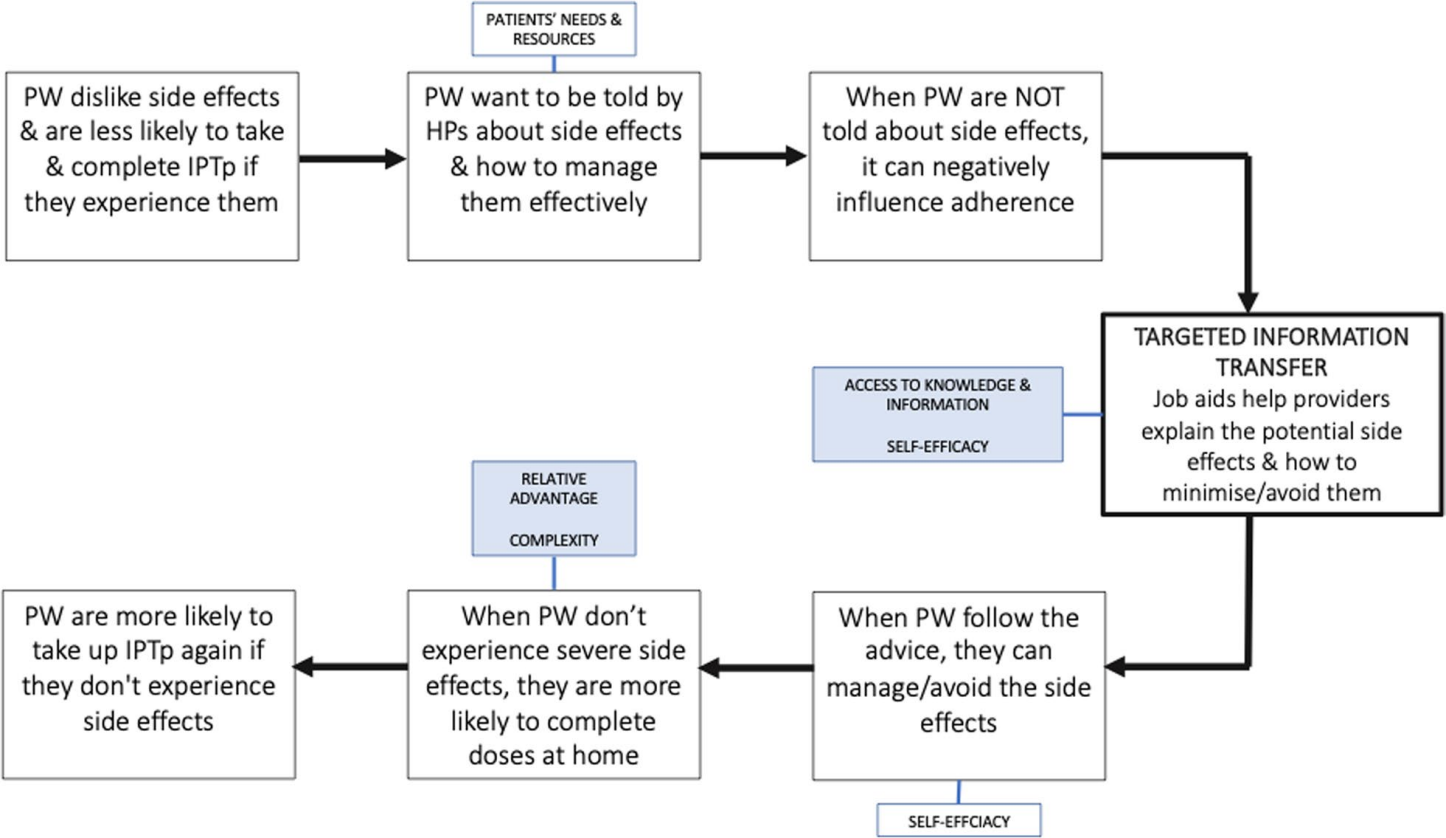
Use of the CFIR constructs to understand how improved adherence to DP doses influenced provider perspectives on IPTp-DP implementation feasibility. Shaded boxes indicate CFIR constructs related to healthcare providers (HP) , unshaded boxes indicate constructs related to pregnant women (PW)

A total of 128 IDIs with healthcare providers (*n* = 64) and pregnant women (*n* = 64) were conducted across 15 facilities in Kisumu and Homa Bay counties. Characteristics of healthcare providers and pregnant women are shown in Table 2. The results presented below describe how and why (1) the targeted information transfer intervention improved adherence by helping women minimize and/or avoid side effects to DP, (2) improved adherence to IPTp-DP influenced provider perspectives on implementing multi-day drug regimens for IPTp using the CFIR constructs of relative advantage, complexity, access to knowledge & information, patient needs & resources and self-efficacy (Fig. 2).

**Table 2:** Characteristics of healthcare providers and pregnant women

Health providers	Intervention group			Pregnant women	Intervention group		
	SP	DP	DP+		SP	DP	DP+
TOTAL INTERVIEWED N=64	22	23	19	TOTAL INTERVIEWED N=64	20	22	22
Female	17	15	11	Married	17	16	14
Male	5	8	8	Single	3	6	8
Average age	36.9	36.4	36.6	Average age	24.7	25.1	23.9
LOCATION				LOCATION			
Kisumu	11	12	10	Kisumu	9	11	12
Homa Bay	11	11	9	Homa Bay	11	11	10
QUALIFICATION				NUMBER OF CHILDREN			
Diploma	18	14	18	0	6	6	9
Higher diploma	1	3	0	1	4	7	8
Degree	2	5	1	2	3	4	3
Certificate	1	1	0	3+	7	5	2
CADRE				EDUCATIONAL LEVEL			
Nursing officer	9	11	12	Primary level incomplete	3	3	5
Nursing in-charge	4	4	2	Primary level complete	4	10	5
Facility in-charge	6	6	5	Secondary level incomplete	3	2	3
Other nurse (MCH, PMTCT, student)	3	2	0	Secondary level complete	7	3	7
TIME AT HEALTH FACILITY				College/diploma	3	4	2
2 Years or less	17	15	9				
More than 3 years	5	8	10				

PW – pregnant women, HP – healthcare provider

### How a targeted information transfer intervention improved adherence to IPTp-DP among pregnant women

Pregnant women reported several unpleasant side effects to DP including nausea, vomiting, fatigue, and dizziness – and suggested that side effects were the main reason some women did not adhere to drug regimens, including with DP. Healthcare providers echoed these concerns and believed that women who experienced side effects were less likely to complete the 2nd and 3rd DP doses at home. The relationship between side effects and non-adherence was reinforced by the few women who reported either skipping doses (e.g., taking a dose every other day) of DP or stopping after one dose (i.e., not taking the DP dose on day 2 or 3) – as they attributed their non-adherence to unpleasant side effects.

*"Interviewer (I): What was your experience with these drugs that made you decide to take them as you skip some days? Respondent (R): I felt tiredness and restlessness and then I decided to start skipping days as I take the drugs."*

Pregnant woman 24, (age 23, 1 child, IPTp-DP+)

*"Personally, my view is that sometimes I take it and it depends on how I am feeling, if I am feeling bad then I will not finish the dose because I know if I continue taking then I must feel the same as the day before. So I would not take it."*

Pregnant woman 1, (age 23, 1 child, IPTp-DP)

*"Maybe this lady takes the drug and starts vomiting and feels dizziness, she will not even take that drug tomorrow or may be if she takes it and experience some pain in the abdomen; she will not take it again."*

Healthcare provider 10, (age 39, certificate, IPTp-DP)

Pregnant women in both IPTp-DP and IPTp-DP + arms clearly articulated their preference for being given information regarding IPTp drugs prior to taking them. This included dosing instructions, the benefits of taking the drugs, and potential side effects they might experience. Some women in the IPTp-DP + arm suggested that information on how to minimize the side effects to DP was particularly useful. Crucially, links between lack of knowledge about potential side effects and reduced adherence to DP were made by both women and providers. That is, knowing about the potential side effects reduced women’s fear and helped them be psychologically prepared, though a minority of women suggested being told about side effects could deter them from taking up IPTp. Despite this clear mandate for information, several women in both IPTp -DP/DP + arms (although fewer in the IPTp-DP + arm) reported being told very little about IPTp.

*"…if you give drugs to somebody and you have not explained to her how the drug works, so I may stop taking it because I am the one who is experiencing the side effects; but if you advise me to continue with it that is how it makes people feel, I will continue taking it."*

Pregnant woman 28, (age 26, 1 child, IPTp-DP+)

*"If you fail to talk of the side effects of the drug and when the mother witnesses it, she will stop taking the drug."*

Pregnant woman 47, (age 26, 1 child, IPTp-DP+)

*“If they take it without knowing the side effects they will default after the 1st dose.”*

Healthcare provider 27, (age 35, diploma, IPTp-DP+)

Providers in the IPTp-DP + arm believed that information regarding side effects to DP and how to manage them had helped women to minimize side effects – including nausea and tiredness – which contributed to their completing the doses at home. This perception was reinforced by women who reported that following the guidance had helped them minimize the side effects. One woman suggested she was able to avoid side effects altogether by taking the drugs at night and going straight to sleep. Interestingly, some women in the IPTp-DP arm reported taking DP doses at night to avoid the side effects, suggesting that either women find ways of managing the side effects themselves or some providers were already giving this advice to women.

*"It [job aid] guides on the side effects what the mother will experience and how to advise. The job aids are very important."*

Healthcare provider 27, (age 35, diploma, IPTp-DP+)

*"As for me I didn’t see any [side effects] because I used to take them at night and went to sleep immediately so if I wake up in the morning I am just okay."*

Pregnant woman 44, (age 26, 0 children, IPTp-DP+)

*"After giving them [advice] on how to take [DP] and how to manage the side effects, they’ve not complained [about side effects]."*

Healthcare provider 24, (age 48, diploma, IPTp-DP+)

*"I: what informs the thought that adherence will improve? R: there is the notion of mothers that SP makes them vomit or feel unwell, but this has not come out with DP, so adherence is likely to be better."*

Healthcare provider 26, (age 36, diploma, IPTp-DP)

Subsequently, some providers observed that at the following ANC visit when asked about their experience with the drugs, women who did not experience side effects were happy to take the next IPTp dose.

*"…But now we started giving them to take in the evening, they are not having any side effects and they are adhering well, they are tolerating. We do make an observation when they come back, we gave you some drugs, how did you go on with it? I didn’t have issues …All our clients that we’ve started with, there is none that have discontinued."*

Healthcare provider 24, (age 48, diploma, IPTp-DP+)

### How improved adherence to DP among pregnant women influenced provider perspectives on implementing multi-day drug regimens for IPTp

Healthcare providers in both the IPTp-DP and IPTp-DP + intervention arms perceived the *relative advantage* of DP over the current drug SP. Specifically, that DP was more effective in protecting women from malaria infections and was better tolerated by women when compared to SP (i.e. women complained of more side effects when taking SP). Conversely, providers in control facilities (IPTp-SP) focused more on the *complexity* of shifting to a multi-day drug regimen. Specifically, they had concerns about not directly delivering all doses by DOT and worried about adherence to DP doses taken at home, mainly based on their perceptions that women often did not complete drug regimens for several reasons, such as side effects, size and smell of tablets, and forgetting to take them.

“I applaud continuing with SP because it is easy to give. You give DOT [directly observed therapy] no drug is carried home; we don’t need any treatment buddy so to me I will say SP to continue…”.

Healthcare provider 20, (age 44, diploma, IPTp-SP)

Interestingly, though providers across all arms acknowledged the challenges that multi-day drug regimens posed in relation to adherence, providers in both IPTp-DP/DP + arms emphasized the advantages of DP over concerns about the complicated dosing regimen – unlike their control site counterparts. This was due to the perception by many providers in the IPTp-DP/DP + arms that most women did in fact complete the DP doses at home and as such adherence was less of a concern. This belief was in part due to the perception that DP elicited fewer side effects, and as such women adhered to the regimen. Crucially, among IPTp-DP + providers, the guidance from the targeted information transfer intervention on how to help women reduce side effects helped them give tangible advice to women, which they perceived to have a positive influence on adherence.

*“…so with DP we usually advise them to take after meals*,* at supper just when they want to go to bed because once you are asleep*,* it is rare that you will get irritated and vomit. So with DP we rarely have case of vomiting unless the mother did not follow the instructions on what we advised her to do”.*

Healthcare provider 25, (age 28, diploma, IPTp-DP+)

"…we also tell them to take the other doses at night so after they have eaten so that by the time they wake up the side effect will be gone."

Healthcare provider 39, (age 30, diploma, IPTp-DP+)

The targeted information transfer (*access to knowledge & information)* equipped providers with useful guidance that increased their confidence (*self-efficacy)* in advising women on how to minimize side effects to DP. Adherence to DP was enhanced because pregnant women were given information (*patients’ needs & resources)* to manage side effects effectively (*self-efficacy)* and adhere to the 3-day dosing regimen. When providers believed that adherence to DP would not be an obstacle, their perceptions focused on the *relative advantage* of IPTp with DP over SP, rather than the *complexity* of the multi-day dosing regimen.

## Discussion

This study explored how a supportive intervention improved adherence to the multi-day drug regimen for IPTp-DP in non-trial settings in western Kenya, from the perspectives of healthcare providers and pregnant women. The findings indicate that job aids equipped providers with useful guidance on how to advise women to manage, or in some cases avoid, the side effects to DP when taking doses at home. In addition, using constructs from the CFIR helped to identify how the targeted information transfer intervention increased healthcare provider confidence that pregnant women would adhere to the 2nd and 3rd DP doses taken at home. Provider perceptions of the relative advantage of DP over SP, including that DP is a more effective preventive drug, were enhanced when their concerns about adherence to DP were reduced, which subsequently contributed to their positive opinion on the feasibility of implementing IPTp-DP. Should IPTp policy shift to DP, or any multi-day ACT, a key consideration for implementation should comprise a communication and training package that includes practical strategies for healthcare providers to help women understand the dosing schedule, manage side effects, and improve adherence alongside information on the advantages of using a more effective preventive drug. The provision of job aids that support the transfer of information from provider to woman, particularly around side effects and how to minimize them, should be included.

Evidence from this study suggests that targeted advice from healthcare providers on how to minimize potential side effects can enhance adherence to DP doses taken at home. This supports the findings from the cRCT that adherence to DP was high and further improved when combined with targeted information transfer (adherence in the DP + arm was 16% higher when compared to women in the DP arm) [20]. An important finding given the dearth of evidence on adherence to multi-day ACT regimens for either treatment or prevention during pregnancy. Side effects are a key barrier to uptake of the current policy (IPTp-SP) [18] and can reduce adherence to anti-malarial drug regimens [24], findings supported by this study. Providers in this study emphasized the importance of effective communication with women about DP, including information on potential side effects and the benefits of taking the drug. Importantly, this aligned with women’s desire to be told about the potential side effects of IPTp drugs and how they could minimize the effects (e.g., by taking with food or at night before bed). Information from providers about the negative consequences of malaria in pregnancy and potential side effects encouraged uptake of IPTp-SP in Tanzania [25] and, in particular, amongst women who feared side effects in Ghana [26]. Poor provider communication, including lack of information about the dosing regimens and side effects, pose a serious threat to uptake and adherence of drugs offered at ANC [27]. In this study, a few participants acknowledged that information about side effects may make some women fearful of taking the drugs – but most believed that knowing about the side effects would improve, not deter, adherence. Further, armed with useful strategies from providers, women were able to effectively manage the side effects at home. Though unexplored in relation to IPTp, the notion that self-efficacy enhances adherence to drug regimens is recognized in HIV literature with regards to antiretroviral therapy. Adherence self-efficacy, defined as ‘one’s confidence in his/her ability to take medication as recommended by medical providers’ [28] was found to mediate the relationship between side effects and adherence among HIV patients in China [29]. The authors noted that adherence self-efficacy, which included patients being able to manage side effects, enhanced their willingness to follow dosing instructions.

Unsurprisingly, healthcare providers in all three arms shared concerns about adherence to the 3-day dosing regimen of DP. However, providers in the IPTp-DP + arm found job aids useful when advising women on how to minimize side effects, which contributed to their confidence in the use of, and adherence by women to, DP. This confidence meant providers focused on the relative advantage of using DP, rather than the added complexities that a multi-day drug regimen presents – including not being able to administer all doses by DOT and concerns about not knowing if women completed doses at home. Providers in both DP arms perceived the drug to be better tolerated and more effective than SP, a finding substantiated in previous studies [12, 13], which suggests that in practice the potential challenges associated with use of DP for IPTp may not play out. But the added value of the supportive intervention was that it enhanced the self-efficacy of providers in relation to adherence, and this could have positive implications for provider acceptability towards multi-day drug regimens for IPTp. Self-efficacy is a central construct of acceptability, described by Sekhon as ‘the participants confidence that they can perform the behaviour(s) required to participate in the intervention’ [30]. Policy makers and implementers looking to shift to IPTp drugs with multi-day dosing regimens should note that targeted information transfer enhanced both provider and pregnant women’s confidence in side effect management and adherence to DP. Further research on how to improve adherence to multi-day drug regimens for IPTp should be conducted if policy shifts to ACTs.

### Strengths and limitations

Interviews with women relied on historical recall and self-reported adherence to the DP doses taken at home on days two and three. As such there could be some inaccuracies in those accounts. Social desirability bias could apply to both healthcare providers and pregnant women who may have responded in such a way as to please the interviewers. In addition, changes to the way IPTp was delivered during the COVID-19 pandemic (e.g., the first dose by DOT was replaced with self-administration of all doses at home in some clusters across all the arms) means that these interventions were assessed in ‘a changed context’. These changes occurred in some facilities across all three study arms which reduces the likelihood of any effects being limited to a single arm. The use of convenience sampling limits the generalisability of the study findings. However, this study is strengthened by the large number of participants included across a range of health facilities, including different healthcare provider cadres, in each trial arm.

## Conclusions

Pregnant women wanted to be informed about potential side effects to IPTp drugs and valued the advice from healthcare providers, as part of a targeted information transfer intervention, on how to minimize them. Both pregnant women and providers believed that effective management of side effects improved adherence to DP doses taken at home. Enhancing pregnant women’s ability to manage side effects to drugs at home and boosting provider confidence by equipping them with the tools and information to guide women, would go a long way in reducing the key concerns around a policy shift from IPTp with single-day SP to a multi-day ACT.

## Supplementary Information

Supplementary Material 1.

## Data Availability

No datasets were generated or analysed during the current study.

## Abbreviations

ACT: Artemisinin-based combination therapy
ANC: Antenatal care
CFIR: Consolidated Framework for Implementation Research
cRTC: Cluster randomized controlled trial
DOT: Directly observed therapy
DP: Dihydroartemisinin-piperaquine
IPTp: Intermittent preventive treatment in pregnancy
ISTp: Intermittent screening and treatment in pregnancy
SP: Sulfadoxine-pyrimethamine
WHO: World Health Organization
