# Supplementary material for: “If they take it without knowing, they will default…”: perceptions of targeted information transfer to promote adherence to intermittent preventive treatment with dihydroartemisinin-piperaquine for the prevention of malaria in pregnancy in western Kenya
Source: Malar J. 2024 Nov 29;23:364. doi: 10.1186/s12936-024-05131-6 (PMC11605956; doi:10.1186/s12936-024-05131-6)
Supplement: Supplementary file 1 — Supplementary Material 1. [file 12936_2024_5131_MOESM1_ESM.docx]

Table S1: The Standards for Reporting Qualitative Research (SRQR) checklist

| **Title and abstract** |  | Page number |
| --- | --- | --- |
| 1. Title | Concise description of the topic of study | P1 |
| 2. Abstract | Includes background (& aim of study), methods, results, conclusions | P2 |
| **Introduction** |  |  |
| 3. Problem formulation | Description of phenomenon being studied, review of empirical work | P3 |
| 4. Purpose or research question | Purpose of the study/specific objectives | P3, P6 |
| **Methods** |  |  |
| 5. Qualitative approach and research paradigm | Qualitative study and rationale for study design | P4 |
| 6. Researcher characteristics and reflexivity | Description of researchers, data collectors | P5 |
| 7. Context | Description of study sites | P4 |
| 8. Sampling strategy | Selection of study sites; healthcare provider and pregnant women selection for in-depth interviews | P4 |
| 9. Ethical issues pertaining to human subjects | Ethics approval from four ethics committees; informed consent from participants | P12 |
| 10. Data collection methods | Description of data collection (in-depth interviews) | P4-5 |
| 11. Data collection instruments and technologies | Details of discussion topic guides | P4-5 |
| 12. Units of study | Number, type and characteristics of participants | P7 |
| 13. Data processing | Management and processing of data | P5 |
| 14. Data analysis | Description of coding and analysis – thematic analysis; framework used to support analysis | P5-6, P15 |
| 15. Techniques to enhance trustworthiness | Description of coding validation discussions and consensus building among the research team | P5 |
| **Results/Findings** |  |  |
| 16. Synthesis and interpretation | Main findings reported | P6-10 |
| 17. Links to empirical data | Figures illustrating syntheses of findings | Figures 2-3 |
| **Discussion** |  |  |
| 18. Integration with prior work, implications, transferability, and contributions to the field | Summary of main findings, situating findings along existing literature; discussion of how these findings contribute to the field of inquiry | P10-12 |
| 19. Limitations | Limitations of the study, generalisability of the findings | P11 |
| **Other** |  |  |
| 20. Conflicts of interest | None to declare | P12 |
| 21. Funding | EDCTP2 programme | P12-13 |
